# A preventive care approach for oral health in nursing homes: a qualitative study of healthcare workers’ experiences

**DOI:** 10.1186/s12877-024-05396-1

**Published:** 2024-10-01

**Authors:** Lisa Bellander, Eva Angelini, Pia Andersson, Catharina Hägglin, Helle Wijk

**Affiliations:** 1 Department of Behavioral and Community Dentistry, Institute of Odontology, Sahlgrenska Academy, University of Gothenburg, 40530 Gothenburg, Sweden; 2 Centre of Gerodontology, Public Dental Service, Region Västra Götaland, 40233 Gothenburg, Sweden; 3 Institute of Health and Care Sciences, Sahlgrenska Academy, University of Gothenburg, 40530 Gothenburg, Sweden; 4 Department of Oral Health, Faculty of Health Sciences, Kristianstad University, 29188 Kristianstad, Sweden; 5 Department of Architecture and Civil Engineering, Chalmers University of Technology, 41296 Gothenburg, Sweden; 6 Department of Quality Strategies, Sahlgrenska University Hospital, Region Västra Götaland, 41345 Gothenburg, Sweden

## Abstract

**Background:**

Oral health problems are common among care-dependent older adults living in nursing homes. Developing strategies to prevent the deterioration of oral health is therefore crucial to avoid pain and tooth loss. A standardized work widely used in nursing homes in Sweden is the quality register Senior Alert (SA), which assesses age-related risks concerning e.g. pressure sores, falls, malnutrition and oral health. The oral health assessment is performed with the Revised Oral Assessment Guide-Jönköping (ROAG-J), which also includes planning and implementation of preventive oral care interventions with the goal of achieving good quality care. However, what facilitates and hinders healthcare workers in working with oral health in SA remains unexplored. The aim of this study was to describe healthcare workers’ experiences of assessing oral health with the ROAG-J, planning and performing preventive oral health care actions in accordance with SA in nursing homes.

**Methods:**

Healthcare workers (*n* = 28) in nursing homes in two Swedish municipalities participated and data was collected through six focus group interviews. Reflexive thematic analysis was used to identify patterns of meaning in the data.

**Results:**

Themes generated in the analysis were:

1. A structured process promotes communication and awareness and stresses the importance of oral health;
2. Oral care for frail older adults is challenging and triggers ethical dilemmas;
3. Unclear responsibilities, roles and routines in the organization put oral health at risk;
4. Differences in experience and competence among healthcare staff call for educational efforts.

**Conclusions:**

The structured way of working increases staff awareness and prioritization of oral health in nursing homes. The main challenges for the healthcare workers were residents’ reluctance to participate in oral care activities and oral care being more complicated since most older adults today are dentate. Organizational challenges lay in creating good routines and clarifying staff roles and responsibilities, which will require continuous staff training and increased management involvement.

**Supplementary Information:**

The online version contains supplementary material available at 10.1186/s12877-024-05396-1.

## Background

Good oral health and regular dental care have made it possible for a large proportion of adults to retain their natural teeth into old age [1]. Retaining teeth and maintaining a good oral health are important for people’s quality of life and general health [2, 3]. However, for those with natural dentitions, the risk of developing oral diseases such as caries, periodontitis and tooth loss increases significantly with increasing age and multimorbidity [4, 5]. In older ages, functional and/or cognitive impairments are common, making it difficult to perform daily oral self-care. In addition, polypharmacy can lead to reduced salivary flow and a fast caries progression [6, 7]. The most care-dependent older adults are those who live in nursing homes in Sweden; many of them have both cognitive and functional problems, and oral diseases are therefore also very common [8].

Oral diseases in the rapidly growing ageing population are a public health problem and there is an urgent need to develop strategies to promote and prevent them [9]. Improving oral health in older adults in nursing homes is complex and there are not yet any proven effective implementation strategies to be recommended [10, 11]. However, since oral diseases are usually preventable, it is important to assess oral conditions regularly to make an early diagnosis and include preventive oral actions in daily practice [12].

Studies have reported that poor oral health affects older adults’ wellbeing and is associated with issues pertaining to pain and problems with eating, swallowing and social interactions [3]. Impaired oral health can also have a negative impact on general health conditions such as cardiovascular disease and diabetes, and it can lead to malnutrition and aspiration pneumonia [13, 14]. Multiple factors influence deteriorating oral health in older adults living in nursing homes [15]. The residents are usually dependent on healthcare providers to help and support them with activities of daily living (ADL). This also applies to oral care to maintain oral health [10]. However, oral care in nursing homes has been shown to be a low priority. Reasons for this include time pressure, high workload as well as negative attitudes and insufficient competence in oral health care among the nursing staff [16, 17]. Difficulties performing toothbrushing due to technically complicated dental replacements such as dental bridges and implants, and non-functioning collaboration with the dental care services are other reported obstacles [15, 18]. In addition, nursing homes usually lack oral health routines such as oral care guidelines and documentation in practice [19, 20]. Studies have also reported that nursing home residents often hesitate to ask for help with daily oral care, and that dental care visits are being significantly reduced for people diagnosed with dementia [21, 22].

**Fig. 1:**
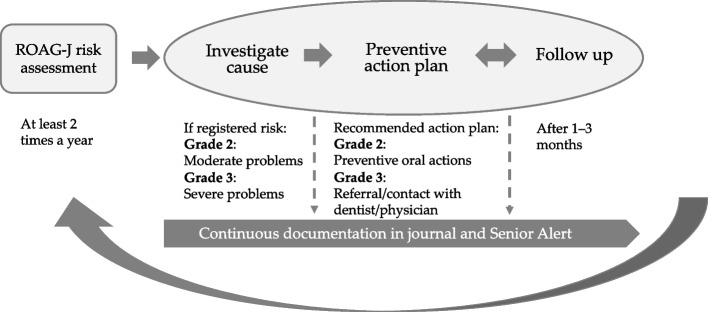
The preventive care approach for oral health in the quality register SA [26]

A possible strategy to detect oral health problems and introduce preventive oral care actions is the Swedish national quality register, Senior Alert (SA), which has been used in e.g. nursing homes since 2008 [23]. The register takes a systematic approach and uses a preventive care process for age-associated risks. Since 2011 it has included prevention of poor oral health. The other risk areas included in SA are falls, pressure ulcers, malnutrition and bladder dysfunction. The preventive care process includes risk assessments, care measures and evaluation of health outcomes. The results can be followed over time at both individual and organizational levels. To assess oral health status in SA, the Revised Oral Assessment Guide (ROAG) is used. The ROAG is designed for use by non-dental professionals and has been validated and shown to have high sensitivity and specificity [24, 25]. The ROAG was slightly modified to be used in SA, and also includes suggestions for preventive oral care actions, and was therefore renamed ROAG-Jönköping (ROAG-J). The ROAG-J assessment is user friendly and includes inspection of the voice, lips, oral mucosa, tongue, gums, teeth, dentures, saliva and swallowing [see Additional file 1]. In the event of a registered moderate or severe change in oral health in ROAG-J, appropriate actions should be implemented and then systematically followed up in SA. The preventive care approach for oral health in SA, with the goal of improving the oral care quality for care-dependent older adults, is shown in Fig. 1.

SA is well established in Sweden and almost all municipalities use the quality register. In 2022, about 88 000 ROAG-J assessments were made for about 64 000 nursing home residents, which constitutes approximately 70% of all older adults living in Swedish nursing homes [27, 28].

Previous national registry-based studies that examined the use of ROAG-J and preventive oral health measures in SA found fewer detected oral health problems and implemented actions than expected. It was discussed that these results could be due to deficiencies in procedures in nursing homes and/or insufficient training in ROAG-J [26, 29]. However, what facilitates and hinders nursing staff in working systematically with preventive oral health care in accordance with SA remains unexplored. Therefore, this study aims to illuminate healthcare workers’ experiences of assessing oral health using ROAG-J, planning and performing preventive oral health actions for older adults in nursing homes.

## Methods

### Design

This study had a qualitative descriptive design [30, 31] which can generate a deeper understanding of healthcare workers’ experiences of working with ROAG-J and oral health in nursing homes. Focus group interviews was used as this method can capture the participants' experiences and be useful for exploring possible group norms and workplace cultures [32]. The consolidated criteria for reporting qualitative research (COREQ) was used [see Additional file 2] [33].

### Setting

The municipalities and regions in Sweden are responsible for planning the development and organization of healthcare and services according to the needs of nursing home residents. All older adults living in nursing homes have support from social services (responsibility of the municipalities) and most of them also from healthcare (responsibility of the regions). Nurse assistants work within social services and registered nurses work within healthcare. Healthcare and social services are based on two different legal acts and separate medical record systems. Registered nurses coordinate and are responsible for the healthcare given to the residents. The nurse assistants in nursing homes usually consist of both trained staff and personnel without formal training. The nurse assistant role is to monitor and care for residents on a daily basis, and they often assist with ADL, such as personal hygiene including oral care. They are to report illness and health risks to registered nurses and can perform healthcare actions and risk assessments in Senior Alert (SA), such as ROAG-J, if delegated by a registered nurse.

The dental care services’ presence in nursing homes varies between municipalities, and there are both private and state-owned dental care providers that offer some domiciliary dental care in nursing homes. In Sweden, there is a reimbursement programme for older adults with extensive care needs, which gives them the opportunity to receive essential and subsidized dental care and an annual screening oral health assessment (not ROAG) free of charge, including oral care advice from a dental hygienist.

### Selection

The participants included in the study were healthcare workers comprising registered nurses and nurse assistants. They were invited from six selected nursing homes in two municipalities in western Sweden using a purposive sampling technique where the participants had experience working with ROAG-J in SA.

The nursing homes selected for this study had been actively working with ROAG-J in recent years, which means that they frequently had both registered assessments and planned and implemented oral care actions in the quality register according to the results available from SA’s public report page [28]. In three of the included nursing homes, dental hygienists had also, within a recently completed special project, developed and carried out a new ROAG training for healthcare workers [34]. This strategic selection was made to be able to capture different experiences and to achieve a breadth in data.

The managers of the selected nursing homes were contacted about the study by email or telephone. They were asked to inform the registered nurses and nurse assistants who performed the ROAG-J about their interest in participating in the study. Contact information of interested healthcare workers was obtained from the managers and written information about the study, including a consent form, was sent to all participants before the interviews. The authors had no previous relationship with the participants. Before the interviews, demographic information about the participants was collected via individual questionnaires.

### Data collection

A total of six focus group interviews were conducted and each group consisted of four to six participants. To avoid hierarchies between professional groups, focus group interviews were conducted separately with registered nurses and nurse assistants, with three interviews for each profession. All interviews were conducted in the participants’ workplace or at another nearby nursing home during working hours. The first author (LB) moderated the group interviews using a semi-structured interview guide with open-ended questions focusing on the healthcare workers’ experiences of assessing oral health with ROAG-J and planning and carrying out preventive oral care actions in accordance with the quality register SA [see Additional file 3]. The interview guide was developed by the authors specifically for this study and was based on the focus area of the research questions. The opening question in all interviews was: “What are your experiences of performing oral health assessments?”. An open dialogue was conducted with follow-up questions that were based on what came up during the interviews. One of the authors (EA) acted as observer and took notes of the verbal and non-verbal communication in all interviews. The data were collected between May and October 2022. The interviews were audio recorded and lasted between 33 and 73 min (mean 53 min) and were transcribed verbatim.

### Data analysis

A qualitative reflexive thematic analysis according to Braun and Clarke was chosen to identify, process and report shared patterns of meaning across the dataset [35–37]. This method was chosen because it provides a theoretically flexible and clear guideline of six phases to ensure rigour. The analysis began with all the authors reading and familiarizing themselves with the content of the dataset, and preliminary initial codes and notes were created. In the second phase, meaningful entities of data and how these answered the research question were identified, organized and coded by LB using NVivo Version R1/2020. In the third phase, conducted by LB, codes with related content were combined to generate initial themes. In the fourth phase, the preliminary themes were reviewed and modified through a dialogue between the authors to make meaningful contributions to answering the research questions and supporting the entire dataset. In the fifth and sixth phase, all authors worked together to describe the meaning of each theme, name final themes and produce the report. The reflexive thematic analysis is a recursive process, with movement back and forth throughout all phases. The members of the research group, who have different knowledge and perspectives in the field, regularly discussed the analysis reflectively throughout the process to ensure the credibility of the results.

## Results

All invited healthcare workers (*n* = 28) participated in the study, of which 14 were registered nurses and 14 were nurse assistants. Participants’ characteristics are presented in Table 1.

**Table 1:** Characteristics of the participants (*n* = 28)

	Registered nurses (*n* = 14)	Nurse assistants (*n* = 14)
**Gender**, female, *n*	14	14
**Age**, mean (range)	49 (26–63)	49 (24–63)
**Clinical experience in years,** mean (range)	19 (1–37)	22 (1–44)
**Years in workplace,** mean (range)	9 (1–22)	9 (1–32)
**Have received oral care training,** *n* (%)	5 (36)	7 (50)
If yes^*^: in basic education/in further education/at workplace	4/0/2	3/1/4
**Have received ROAG-J education,** *n* (%)	8 (57)	9 (64)
Training by dental professionals/web-based education^*^	6/2	5/4
Years since last education, mean	5	6

^*^Multiple response options were possible

In the analysis of the data, patterns were identified and categorized into four themes that illustrate the healthcare workers’ experiences of assessing oral health using ROAG-J and performing oral care for older adults in nursing homes:

*- A structured process promotes communication and awareness and stresses the importance of oral health;*
*- Oral care for frail older adults is challenging and triggers ethical dilemmas;*
*- Unclear responsibilities, roles and routines in the organization put oral health at risk;*
*- Differences in experience and competence among healthcare staff call for educational efforts.*

Quotes are used to illuminate the findings for each theme.

### A structured process promotes communication and awareness and stresses the importance of oral health

The healthcare workers regularly performed ROAG-J with the other risk assessments in SA. They described it as important, as the general and oral health status of the residents often gradually deteriorated. Many felt oral health to be an important part of everyday care and that oral health problems are linked with other risks in the SA register, such as poor oral health leading to difficulty with nutrition and weight loss.

*“You have to think it all the way through ... You can’t chew if you don’t have teeth.”*

*(Nurse assistant)*

The oral health assessment was either done separately by the registered nurses or the nurse assistants, or by both jointly. Some of the nursing homes had a ready-made “ROAG-box” with mouth mirror, flashlight and assessment cards, which made it more feasible to carry out the assessments.

The registered nurses felt that performing the ROAG-J clarified what aspects to inspect within the oral cavity and increased the chances of not missing oral health problems. Problems discovered could therefore easily be raised with a physician or a dentist. They also found it easier to describe in the medical records what was discovered than if no assessment were made.

*“It is easier to make an assessment today [using ROAG-J] than having to look and describe it yourself [in the medical records]. So, it has become easier in a way.”*

*(Registered nurse)*

The healthcare workers found that assessment using ROAG-J was important because the oral cavity is not visible from the outside, meaning that one may not notice, for example, whether the teeth are brushed or not. Another reason mentioned was that many residents were unable to express experience of pain or problems.

*“But sometimes they [the residents] have had something wrong with a tooth, or they’ve had an abscess, or you can see that they have a toothache, but they can’t express it.”*

*(Registered nurse)*

Some of the registered nurses thought it would be good if nurse assistants could do the ROAG-J, as they were close to the residents and therefore could capture the older adults in everyday situations. This was confirmed by the nurse assistants, who said they could detect oral health risks by noticing if the residents ate less, if it hurt when brushing their teeth, or if they grimaced.

*“You have to keep an eye out … To see if someone who used to eat very well suddenly doesn’t eat … Maybe someone is sitting there in pain … – ‘I don’t want to eat it’ – even if they can’t tell me about it.”*

*(Nurse assistant)*

The risk assessments in SA were often made before the monthly team meetings, where each older adult’s health situation was brought up for discussion at least every six months. Those who attended the team meetings were often registered nurses, nurse assistants, unit managers and rehabilitation staff. The healthcare workers experienced the meetings as positive, as the team discussed the older adults’ risks, especially as they could have different views. The meetings made it possible to discuss each older adult and their individual needs in depth. At the meetings, the nurse assistants usually described oral care actions taken and discussed whether residents took care of their oral hygiene themselves or needed help from the staff.

*“Well, it’s the whole person we discuss there [at the team meetings] … I think it works very, very well. And that very much includes the mouth. We’re talking about the whole person, after all.”*

*(Nurse assistant)*

The assessments with identified risks and planned actions were recorded in SA, which enabled all healthcare workers to see the results at the team meetings. They were also used in the SA follow-ups carried out after three months. The healthcare workers expressed that they sometimes could distinguish improvements in health outcomes, including oral health status, at the follow-ups.

*“When we do a follow-up ... We did this planning in Senior Alert ... You can see that it has improved. Yes, so you can see the result most of the time.”*

*(Registered nurse)*

#### Oral care for frail older adults is challenging and triggers ethical dilemmas

The healthcare workers described facing several contextual challenges in maintaining good oral health and performing ROAG-J. The main challenge they experienced was the residents’ hesitation to cooperate. The older adults could be reluctant to open their mouths because they did not understand, experienced discomfort and pain, or because they felt that their integrity was being violated. Engaging and collaborating with the residents could be time consuming depending on the older adult’s mood. The healthcare workers tried to adapt and could choose to wait and return later in the day. Some healthcare workers also perceived the oral care tasks to be unpleasant. They struggled with the ethical balance between accepting the older adult’s reluctance to participate, their own feelings of discomfort, and doing what is best for them.

*“Sometimes I can find that it becomes very intimate and unpleasant to look closely at someone else’s mouth [with ROAG-J]. But then you have to do it for the other person’s good.”*

*(Registered nurse)*

Most residents had natural teeth and dentures were perceived to be rare. The healthcare workers found that older adults who had retained their natural teeth often had poorer oral health due to the challenge of obtaining their permission to get access to the oral cavity.

*“Well, you have your own teeth. Because of that, many older adults may not allow you to help them, either … When I end up in a [nursing] home, I will have dentures.”*

*(Registered nurse)*

Performing oral care and conducting ROAG-J were perceived as risky as the residents could suddenly clench their teeth. One nurse assistant described that iron gloves would be needed to protect oneself when performing oral care. They were also concerned that parts of teeth could come loose and that the older adult might swallow it or choke. Several of the healthcare workers also expressed that it was practically and technically difficult to perform oral care on the residents. Cleaning with interdental brushes was considered a particularly difficult task.

*“Precisely when you have to go in [with interdental brushes] when there are dental bridges. Yes, it’s very difficult with all these interdental brushes. It’s not even easy to do on yourself.”*

*(Nurse assistant)*

The greatest difficulties were seen for older adults with cognitive impairment. They were often unable to brush their teeth themselves and the nurse assistants needed to help them, although they often refused help. One nurse assistant described that performing oral care sometimes felt like abuse when having to force them to participate. Due to dementia, they could have difficulty understanding the meaning of oral care and thereby easily become worried and defend themselves by for example fighting and spitting.

*“I’ve always thought it was a bit complicated to do regular oral care for our residents with dementia ... Some days you can hardly suggest brushing their teeth, then you’re not worth a dime ... It’s a bit tricky, figuring out how to squeeze in a little toothbrushing.”*

*(Nurse assistant)*

Even residents without cognitive impairment sometimes hesitated to participate in oral care or ROAG-J for reasons of privacy, regardless of whether or not they understood its meaning. They attended to their own oral hygiene, and the healthcare workers therefore had no knowledge of their oral health.

“*It’s private and it’s a violation of their privacy to impose oneself on them. So, it’s quite often that I get that response at the somatic unit*.”

*(Registered nurse)*

The healthcare workers expressed that personalized adaptations and strategies could help to overcome the older adults’ reluctance to participate in oral care and ROAG-J. For example, different sizes of toothbrushes or different flavours of toothpaste could be used. The older adults could better understand that they should open their mouth if they were shown a toothbrush, were stroked on their cheeks, or if the healthcare worker said they were a dentist and wore a face mask.

Another challenge concerning the oral health of older adults that was addressed was that the food for the residents often contained a lot of sugar which some of them needed for energy intake, but which increased the risk of dental caries.

“So, if you [the resident] don’t want to eat lunch or dinner, [maybe] you will be eating sugar, candy all the time."

*(Nurse assistant)*

They expressed it was ethically difficult to justify a diet based on less sugar as relatives often brought sweets, and many older adults had their own fridges and chose independently what they wanted to eat and drink.

### Unclear responsibilities, roles and routines in the organization put oral health at risk

Several healthcare workers mentioned that oral care often was deprioritized to other daily duties at the nursing home. This was due to both difficulties getting the residents to participate in oral care and uncertainty about which professionals would be responsible for their oral health.

*“Oral care falls away in some way, or dental care itself. Because it’s a different profession ... it’s dentists and dental hygienists ... So, it’s not really the expertise we have.”*

*(Registered nurse)*

The registered nurses considered their main responsibility to be in healthcare and they had little insight into the daily oral care routines. They expressed that staff shortages and time constraints made it difficult for them to take on more responsibilities. In palliative care and particularly in end of life care, the participation and responsibility for oral care increased among registered nurses, and they also tended to perform ROAG-J more regularly then.

Nurse assistants, on the other hand, were the ones who supported the older adults in their daily lives and helped with personal hygiene, including daily oral care. The most common oral care routine they described was that they helped with toothbrushing twice a day.

However, the healthcare workers thought that the nurse assistants’ unit managers did not prioritize oral health and did not believe that oral care takes any time to perform. Greater participation and commitment from the managers were requested for oral care to function well.

*“But they are the ones first of all, the unit managers, who should be involved, but they are not … It’s probably not [what] they think about in the first place, that oral health is so important.”*

*(Registered nurse)*

It emerged that there were different practices in the performance of ROAG-J. One example mentioned was that the assessment was not carried out with the older adults present, but only through a discussion between registered nurses and nurse assistants. Sometimes the instruments necessary to perform ROAG-J reliably, namely a flashlight and a mouth mirror, were lacking and some nurse assistants had even needed to purchase their own flashlights.

A competitive situation was also perceived regarding the annual oral health assessment (not ROAG-J) performed in nursing homes by dental hygienists from the dental care services. This made the healthcare workers feel unsure about which oral health assessment they should base the oral care on. Their opinion was that it would be better if the dental professionals could do ROAG-J in SA, as the healthcare workers felt unsure if they were performing the assessments correctly.

*“This is what we also react to, the staff [nurse assistants] as well, that when they [the dental care professionals] come and do an oral health assessment, then it does not count as a ROAG-assessment. And we’ve been thinking that it’s a shame. Because they, the ones who come [here] and are educated, they know this better than anyone else.”*

*(Registered nurse)*

Some registered nurses described the register SA as rigid, in the sense that they knew the residents’ problems before making the assessments or, conversely, that they assessed risks even though they did not perceive the older adult to have any. Routines for oral care actions included in SA were also perceived as unclear, for example that dentists should be contacted when a severe risk was assessed in ROAG-J, which is something they usually did but rarely recorded in the register. There were also some deficiencies in the management of patient recording; for example, double administration in multiple record systems was required when working with SA. The registered nurses also described that they did not have access to read the nursing care records and that the nurse assistants did not routinely read the medical records.

### Differences in experience and competence among healthcare staff call for educational efforts

Several healthcare workers mentioned experience and training as important factors in becoming more confident in performing ROAG-J. Cooperating and helping each other were considered beneficial for being able to obtain reliable assessments. The need to perform several oral health assessments to be able to feel comfortable and safe in the situation was stressed.

“*Because that’s how it is, when you look at several mouths and don’t get scared, then you can be sure of your assessments*."

*(Registered nurse)*

The registered nurses stated that several nurse assistants had difficulty assessing oral health and understanding the content of ROAG-J. It was therefore important for the registered nurses to identify experienced nurse assistants to perform ROAG-J because otherwise they had difficulty trusting the accuracy of the assessments. In line with this experienced nurse assistants were also expected to train newly employed staff in ROAG-J and to inform them about the older adults’ current oral care routines.

*“I have tried to pick the one [nurse assistant] at the unit who I feel is very experienced, skilled, or who is good ... so that I have someone with experience I can inform.”*

*(Registered nurse)*

The composition of healthcare workers, especially the group of nurse assistants, was not homogenous in care experience, as several of the staff were temporary employees. The healthcare workers expressed that it was important that all staff understood the importance of oral health and that it is part of nursing care. Temporary staff sometimes lacked sufficient knowledge and skills in oral care routines, which caused frustration among the permanent staff when the safety of care was compromised.

*“I can say that unfortunately some of the staff may not understand the importance of, how important it is to help them [the residents] … This thing about bringing in hourly employees … I get a little angry sometimes.”*

*(Nurse assistant)*

The registered nurses described that it was more common today that the groups of healthcare workers were multicultural. Variation concerning oral care habits relating to cultural differences, such as how often you brush your teeth, was mentioned by some as a hindering factor for functioning oral care routines at work.

Many healthcare workers described a lack of oral care knowledge provided both in their basic education and at the workplace, and several lacked training in performing ROAG-J. The registered nurses mentioned that all staff needed more continuous training in ROAG-J and oral care, both because they work more frequently with oral health and because of high staff turnover.

*“Two or three days may pass before they [the residents] get their teeth brushed, until someone [a nurse assistant] comes who has learned it or has it as a routine. Because I think that more education, more support out there [is needed].”*

*(Registered nurse)*

## Discussion

The present study has investigated the experiences of healthcare workers in assessing oral health using ROAG-J and performing preventive oral health actions in nursing homes within the Senior Alert (SA) quality register. We discovered both facilitating and hindering factors for the staff in working systematically with preventive oral care.

### A structured process promotes communication and awareness and stresses the importance of oral health

The results revealed that working in a structured way increased the healthcare workers’ focus on oral health, which thus received a higher priority in nursing homes. It emerged that the participants had a holistic perspective where they considered oral health significant for the residents’ overall health and wellbeing. Additionally, as oral health is an integral part of the SA register, it becomes a more important part in routines and healthcare workers’ daily tasks. The working model for the preventive care approach in SA consists of assessments, diagnosis, planning, implementation and evaluation, which also are the phases described in the nursing process that should contribute to good nursing quality and prevent missed nursing care [38]. Previous research has expressed that the reason for neglect of oral care in nursing homes may be the difficulty for older adults to express oral health problems unless they cause pain, and that problems in the mouth are not immediately visible to the staff [15, 39, 40], which the participants in the present study also stated. This highlights the importance of examining residents’ oral health at enrolment and routinely thereafter [12, 19, 41]. The systematic assessment of oral health provided by ROAG-J also made it clear to the participants which parts of the oral cavity to inspect. Abnormalities in the oral cavity can be detected at an early stage and actions can be taken before problems are caused and to prevent unnecessary suffering for the older adults. Noguchi et al. have also highlighted the benefit of performing systematic oral health assessment with ROAG in clinical practice, as it helps to detect risk of aspiration pneumonia in older adults [42].

The results showed that through cooperation and teamwork, good routines around oral health could be established. The registered nurses and nurse assistants assisted each other in the assessment of oral health and they discussed and planned preventive actions in the regular SA team meetings. Teamwork and personal interaction within SA have been shown to facilitate preventive work through enhanced learning and work towards a common goal [38, 43–45]. The results of the present study emphasize the importance of communication and regular assessment of oral health in nursing homes and how a structured process such as SA can promote prioritization and awareness of oral health among nursing staff.

### Oral care for frail older adults is challenging and triggers ethical dilemmas

The results also uncovered contextual limitations in assessing oral health and performing oral care. Individual barriers surrounding the nursing home residents emerged. Many older adults retain their natural teeth, which poses a challenge to the healthcare workers when inspecting the oral cavity and performing daily oral care. Brushing teeth was difficult, time consuming and could even be experienced as repulsive, as other studies also have confirmed [46, 47]. The difficulty of cleaning teeth in the ageing population, often with complex prosthetic constructions, poses a great risk for older adults who are not functionally or cognitively capable of performing their own daily oral hygiene, as they risk experiencing decline in oral health. They need to receive the necessary help for this task, which places high demands on nursing staff being sufficiently trained in oral health and oral care to be able to assist them. Residents’ reluctance to cooperate during oral health assessments and oral care due to cognitive decline and safeguarding their integrity at the same time was seen as the biggest challenge, which also has been confirmed by other studies [15, 16, 46, 48]. In addition, older adults in general are often hesitant to ask for assistance with oral care [49]. Respecting the residents’ autonomy and not forcing them against their will versus carrying out the necessary oral care is a difficult balancing act for the staff [17, 50]. These are examples of ethical challenges that were found in the present study. However, if the healthcare workers were experienced and had a good relationship with the resident, individually adapted, person-centred behavioural strategies could help them carry out the oral care activities despite the older adults’ reluctance. This underlines the importance of nursing staff being trained and working closely with residents to be able to help them with their oral health. However, there is little research on interventions to improve oral care that also support residents with behavioural problems and dementia [10].

Another challenging situation that arose in the present study was the residents’ frequent intake of fast carbohydrates containing sugar, such as candy, snacks, sweetened beverages and nutritional drinks. This problem is relevant for both present and future ageing populations, since the majority of older adults are dentate and caries development is based on a high and frequent sugar intake. Dietary changes with a reduction of sugar intake are necessary to prevent caries, but this can be difficult in a care-dependent ageing population as for example nutritional drinks can be essential for them to maintain their weight. Ástvaldsdóttir et al. discussed that it is important to consider the effects of dietary supplements and special food regimens on oral health before introducing them [5]. Other efforts, for example increased fluoride supplementation, are therefore necessary in caries prevention if these dietary changes are needed.

### Unclear responsibilities, roles and routines in the organization put oral health at risk

Structural and organizational obstacles in the nursing home environment were other barriers that were captured by the results. It emerged from some of the participants that there was a lack of established routines for ROAG-J in SA, and it was particularly unclear how the oral health assessment should be carried out. Kalisch et al. described the availability of material resources and labour resources such as education and experience of the nursing staff as antecedents that either facilitate or hinder practice in nursing organizations [38]. Some healthcare workers in the present study perceived that the organization did not prioritize oral health and therefore did not provide them with training in ROAG-J and the materials needed to enable reliable assessments, i.e. mouth mirrors and flashlights. In a previous register-based study, only about one in ten residents with severe problems according to ROAG-J received a referral or contact with a dentist, despite this being the recommended action [26]. However, the participants in this study stated that they often contacted dental care services in case of severe divergences in ROAG-J but did not routinely enter it in the computer-based SA register. If problems and performed actions are not being registered correctly, the register data will not be fully representative of reality. We share the conclusions from the study by Lannering et al., namely that with different local routines, the risk assessments in SA may lack reliability, and the data quality in SA can therefore be questioned [44].

The results revealed there was a lack of clarity regarding the professionals’ specific roles and responsibilities for the residents’ oral health. The nurse assistants took on the biggest responsibility in terms of the preventive oral care in nursing homes. The registered nurses did not feel that they were that involved and wished that the dental care services could take on more responsibility. However, when the residents were at the end of life, the registered nurses were more involved in both the performance of assessments and in daily oral care, which is also seen in other Swedish studies [16, 48]. However, we think that the work in the quality register SA has clarified registered nurses’ responsibilities regarding oral health. They are responsible for performing the oral health assessments, either by themselves or through delegation to the nurse assistants, and usually also for recording the results in the SA register. In addition, they participate in team meetings where results are discussed on an individual level and preventive actions are planned. Overall, this made the registered nurses more familiar with the residents’ oral health.

The nurse assistants’ managers are responsible for maintaining a high quality of care in nursing homes. However, the participants felt that the managers did not prioritize oral health as they did not give them the right conditions, such as time to perform oral care activities. Not taking explicit responsibility for oral health at management level was also reported by Lindqvist et al. [46]. Committed leadership is crucial for creating good conditions for healthcare workers to be able to carry out preventive work [45].

Another disadvantage found was that dental care professionals were not part of the multidisciplinary team and preventive work process outlined by SA, and they did not participate in team meetings. In addition, registered nurses, nurse assistants and dental staff work under different laws and regulations, which complicates responsibilities and roles as well as collaboration between these professions. Also, a separate oral health assessment (not ROAG) called “oral care cards” is carried out annually by dental hygienists at nursing homes. Different oral health assessments in nursing homes confuse the staff and create difficulties regarding the planned preventive oral care. A study by Persson et al. also showed that these oral care cards have not yet been fully integrated in nursing home environments [51]. We suggest that cooperation using the same tools and systems, such as SA, in dentistry and nursing care would emphasize interprofessional collaboration and ultimately improve the oral health of older adults.

### Differences in experience and competence among healthcare staff call for educational efforts

The results showed that the nurse assistant groups consisted of staff with large divergences in competence and experience in the field of oral health. Contributing barriers that could affect residents’ oral care routines were high staff turnover, temporary employees as well as cultural differences. The participants pointed out that these obstacles required an increased effort in continuous and extended education in oral health for all healthcare workers, which also has been highlighted previously [40, 47, 52].

Some nurse assistant participants in the present study had the role as “oral health representative” in their respective nursing home units. Oral health representatives are supposed to have increased competence in the area and thus be able to help untrained or new staff. This is a good idea given the high staff turnover in nurse assistant groups. Studies by Wårdh et al. also found that using specially trained oral health representatives in nursing home units also could promote communication between the nursing and dental professions [53] and that more residents received help with their oral hygiene [54]. However, in the present study, the nurse assistants who were oral health representatives felt that this role was not specified by the managers, and they often lacked the extended skills in oral care required to undertake it.

The participants pointed out that they had a hard time understanding the content of ROAG-J, and that the assessment too often was performed by healthcare workers without sufficient training. Less than half of the participants in the study had been trained in ROAG-J by dental professionals. A six-minute digital training session in performing it on the SA website was otherwise the only training available. Studies have shown that performing the ROAG requires that the healthcare staff are well trained in the instrument to obtain a reliable result [24, 25]. Without any training, the reliability of the ROAG has been shown to be poor or fair among nurses [55]. In a previous registry-based study, we discussed the finding that the low rate of oral health problems detected with ROAG-J in nursing homes nationally could be due to staff being untrained [29]. The healthcare workers experience in the present study triangulate these previous findings. We would therefore like to stress how important it is that when implementing ROAG in nursing home contexts, management needs to provide continuous training sessions by dental professionals for its users in order for them to perform it correctly. The main challenge for the healthcare workers was the residents’ reluctance to participate in the oral care activities. Therefore, dental care professionals that educate healthcare workers need to provide not only individual oral care advice but also training with consideration of behavioural problems.

## Strengths and limitations

Focus group interviews were appropriate in this in-depth research study to obtain detailed responses and because they have an interactive format where participants have the opportunity to freely develop their thoughts and share experiences and knowledge, which enriched the collection of data [32]. We used a purposive selection of participants who worked with ROAG-J and oral health in the quality register SA because they could provide specific knowledge and develop experiences regarding the study’s areas of interest. The participants also came from two different municipalities with varying organizational conditions, and they had varying ages, experience and education regarding oral health and ROAG-J, which increases the strength of the study. A limitation of the study is the lack of gender diversity. However, since the vast majority of both registered nurses and nurse assistants are women, gender diversity was difficult to achieve.

To increase credibility, data analysis was carried out in close collaboration within the research group and followed a coherent procedure according to the analysis method. Extended quotations were presented in relation to the results to enable readers to assess trustworthiness. Another strength was that the research group has backgrounds in both healthcare and dentistry, with different preconceptions, and was therefore able to understand the dataset from different perspectives. By providing an increased understanding of facilitators and barriers that affect oral health work, the results and knowledge from this study are valuable for organizations that care for older adults.

A limitation is that the result was not member checked due to logistical challenges of asking the participants from the six selected nursing homes to validate the content. However, the COREQ checklist was used to enhance and assess the quality of the research conducted. Another limitation of the study was the lack of reflections from managers and dental professionals, as their roles, responsibilities and priorities in working with oral health in nursing homes appeared unclear. Further studies regarding their perspective on the oral health of care-dependent older adults in nursing homes would be desirable.

## Conclusions

Healthcare workers’ assessment of oral health and planning and implementing preventive oral care actions involves a structured approach that emphasizes communication, awareness and the importance of oral health in nursing homes. However, this work is jeopardized by resident-specific difficulties as well as unclear organizational routines and a lack of clarity regarding staff roles and responsibilities. Strategic work to maintain residents’ oral health also requires commitment from managers and continuous staff training in oral health and oral care.

## Supplementary Information

Supplementary Material 1.

Supplementary Material 2.

Supplementary Material 3.

## Data Availability

The data that supported the findings in the present study are available from the corresponding author upon reasonable request.

## Abbreviations

SA: Senior Alert
ROAG-J: Revised Oral Assessment Guide-Jönköping
ADL: Activities of daily living
ROAG: Revised Oral Assessment Guide
COREQ: Consolidated criteria for reporting qualitative research
