# Supplementary material for: A preventive care approach for oral health in nursing homes: a qualitative study of healthcare workers’ experiences
Source: BMC Geriatr. 2024 Oct 1;24:803. doi: 10.1186/s12877-024-05396-1 (PMC11443800; doi:10.1186/s12877-024-05396-1)
Supplement: Supplementary file 1 — Supplementary Material 1. [file 12877_2024_5396_MOESM1_ESM.docx]

| Items | Grade 0 | Grade 1 | Grade 2 | Grade 3 |
| --- | --- | --- | --- | --- |
| Voice | Not applicable to judge | Normal | Dry, hoarse, smacking | Difficulty speaking |
| Lips | - | Smooth, bright red, moist | Dry, cracked, sore corners of the mouth | Ulcerated, bleeding |
| Mucous membranes | - | Bright red, moist | Red, dry, or areas of discoloration, coating | Wounds with or without bleeding, blisters |
| Tongue | - | Pink, moist with papillae | No papillae, red, dry, coating | Ulcers with or without bleeding, blistering |
| Gums | No gums, only mucous membranes | Light red and solid | Swollen, reddened | Spontaneous bleeding |
| Teeth | No natural teeth | Clean, no visible coating or food debris | Coating or food debris locally | Coating or food debris generally, broken teeth |
| Dentures | No dentures | Clean, functioning | Coating or food debris | Not used or malfunctioning |
| Saliva | - | Runs freely | Runs sluggishly | Does not run at all |
| Swallowing | Not applicable to judge | Unimpeded swallowing | Minor swallowing problems | Pronounced swallowing problems |

Additional file 1. ROAG-J (Revised Oral Assessment Guide - Jönköping) in Senior Alert; the items and the grades^1^.

*^1^Grade: 0 = not relevant to assess, 1 = healthy or normal condition, 2 = moderate change or divergence, 3 = severe changes or divergences.*
