# Supplementary material for: A preventive care approach for oral health in nursing homes: a qualitative study of healthcare workers’ experiences
Source: BMC Geriatr. 2024 Oct 1;24:803. doi: 10.1186/s12877-024-05396-1 (PMC11443800; doi:10.1186/s12877-024-05396-1)
Supplement: Supplementary file 3 — Supplementary Material 3. [file 12877_2024_5396_MOESM3_ESM.pdf]

Interview guide for healthcare workers

The key questions guide the focus group interview, supported by follow-up questions when necessary.

**Introduction**	- *Presentation of moderator, observer and group participants*<br>- *Brief information about the aim of the study, the focus group interview method and how it will be conducted (recorded interview)*<br>- *Information about the consent form and the individual questionnaires*
**Opening questions/**<br>*Follow-up questions*	**What are your experiences of performing oral health assessments?**<br>- *Can you describe an oral health assessment situation?*<br>- *Do you experience any* ***difficulties*** *or* ***advantages*** *to perform the oral health assessments?*<br>- *What are your experiences with ROAG and the other risk assessments in Senior Alert?*<br>**How do you perceive that the older adults reacted during the oral health assessment?**<br>- *How is the participation and commitment of the older adults?*
**Key questions/in-depth themes/**<br>*Follow-up questions*	**In what way are you involved in ROAG and preventive oral care actions?**<br>*Please clarify with concrete examples from your everyday work situations.*<br>- *When is the oral health assessment performed?*<br>- *How do you plan and carry out oral care actions?*<br>- *How do you proceed when the older adults are assessed to need a dental care consultant or dental care visit?*<br>- *How confident do you feel performing oral health assessments and oral care actions?*<br><br>**Have your view of oral health and oral care changed after you started working with ROAG and Senior Alert?**<br>- *If Yes: In what way?*<br>- *What do you think is the reason for this?*<br>- *If No: why not?*<br>- *Suggestions for improvement?*<br><br>**What are your experiences of having completed the ROAG training?**<br>*- How have it affected your assessments and work with oral health?*<br>*- Suggestions for improvement?*
**Final question**	**Do you have anything more to add concerning older adults' oral health or about the oral health assessment ROAG?**
